# Supplementary material for: Human West Nile Meningo-Encephalitis in a Highly Endemic Country: A Complex Epidemiological Analysis on Biotic and Abiotic Risk Factors
Source: Int J Environ Res Public Health. 2020 Nov 8;17(21):8250. doi: 10.3390/ijerph17218250 (PMC7664930; doi:10.3390/ijerph17218250)
Supplement: Supplementary file 1 [file ijerph-17-08250-s001.pdf]

International Journal of
Environmental Research and Public Health

MDPI

Table S1. Spearman's correlation coefficient results between the number of human WNND cases for each year (2015–2019) and the abiotic predictors.

Predictor	Value	2015	2016	2017	2018	2019	2015–2019
Average altitude (m)	Rho	−0.07496351	−0.1286847	−0.1242232	−0.1607161	−0.1223553	−0.2449468
	S	5.794e+09	6083571849	6059524679	6256220288	6049456751	6710220813
	p	2.28e-05	3.076e-13	1.975e-12	<2.2e-16	4.219e-12	<2.2e-16
Annual average humidity (%/100)	Rho	−0.04016418	−0.1255573	−0.0876694	−0.1411441	−0.1053015	−0.2155756
	S	5606449529	6066715699	5862501034	6150728048	5957537175	6551911258
	p	0.02339	1.14e-12	7.193e-07	1.206e-15	2.554e-09	<2.2e-16
Surface of flowing water bodies (ha)	Rho	0.04504388	0.01898325	0.003637584	0.03657402	0.03322832	0.05754027
	S	5147180988	5287646876	5370359490	5192833206	5210866417	5079825869
	p	0.011	0.2841	0.8374	0.03899	0.06075	0.001157
Surface of still water bodies (ha)	Rho	0.08180145	0.1181831	0.09991431	0.1275815	0.09129224	0.1841338
	S	4949058927	4.753e+09	4851431213	4702305818	4897903882	4397490822
	p	3.779e-06	2.207e-11	1.59e-08	4.901e-13	2.447e-07	<2.2e-16
Average temperature in spring (March to May) (degree C)	Rho	0.07441398	0.1013378	0.1162005	0.1599574	0.08876406	0.2230996
	S	4988877140	4843758752	4763649060	4527801055	4911530675	4187466876
	p	2.617e-05	9.896e-09	4.748e-11	<2.2e-16	5.216e-07	<2.2e-16
Average temperature in summer (June to August) (degree C)	Rho	0.07388091	0.1189022	0.1239292	0.1588872	0.1147463	0.2394872
	S	4991750382	4749086975	4.722e+09	4533569309	4771487373	4099138126
	p	2.99e-05	1.666e-11	2.227e-12	<2.2e-16	8.263e-11	<2.2e-16
Average of maximal temperature for summer (degree C)	Rho	0.07550537	0.1111877	0.1257269	0.1541404	0.09210693	0.2263494
	S	4.983e+09	4790668297	4712302311	4559154465	4893512741	4.17e+09
	p	1.987e-05	3.114e-10	1.063e-12	<2.2e-16	1.909e-07	<2.2e-16
Average of annual precipitations (mm/ sq m)	Rho	−0.04833897	−0.1211648	−0.08098068	−0.1395484	−0.1062587	−0.2158519
	S	5650511356	6.043e+09	5826449065	6142127003	5962696560	6553400282
	p	0.006353	6.804e-12	4.725e-06	2.524e-15	1.828e-09	<2.2e-16

Table S2. Spearman's correlation coefficient between the number of human WNND cases for each year (2015-2019) and the biotic predictors.

Predictor	Value	2015	2016	2017	2018	2019	2015-2019
Human population	Rho	0.08695938	0.1313478	0.1142014	0.1550048	0.09456008	0.1976989
	S	4921257834	4.682e+09	4774424358	4554495319	4880290354	4324375586
	p	8.843e-07	9.824e-14	1.015e-10	<2.2e-16	8.927e-08	<2.2e-16
Horse population	Rho	−0.002917551	0.006935175	−0.02885493	0.04544007	−0.0138631	0.04435366
	S	5405691447	5352585587	5545493051	5.145e+09	5464687573	5150901255
	p	0.8692	0.6956	0.1034	0.01031	0.4341	0.01229
Fowl population	Rho	−0.004626605	−0.005662356	−0.01842264	0.03663461	0.006818843	0.05233439
	S	5414903191	5420485851	5489263347	5192506652	5353212611	5107885392
	p	0.7941	0.7494	0.2986	0.03867	0.7004	0.003128
Sparrow probability	Rho	0.082925	0.1070365	0.08433186	0.119439	0.05264705	0.1447979
	S	4.943e+09	4.813e+09	4935420072	4746193840	5106200140	4609510424
	p	2.774e-06	1.39e-09	1.873e-06	1.349e-11	0.002953	<2.2e-16
Dove probability	Rho	0.08603096	0.1110499	0.1126401	0.1471729	0.100604	0.2005501
	S	4926261993	4791410771	4782839439	4596709025	4847713899	4.309e+09
	p	1.156e-06	3.275e-10	1.821e-10	<2.2e-16	1.265e-08	<2.2e-16
Jackdaw probability	Rho	0.09021162	0.1258868	0.1468455	0.1887874	0.1240781	0.2515709
	S	4903728371	4711440534	4598473546	4372408180	4721189170	4.034e+09
	p	3.39e-07	9.947e-13	<2.2e-16	<2.2e-16	2.096e-12	<2.2e-16
Crow probability	Rho	0.06464558	0.08531506	0.1004867	0.06913087	0.09111964	0.1338824
	S	5041528452	4930120660	4848346093	5017352930	4898834199	4668344272
	p	0.0002609	1.418e-06	1.315e-08	9.414e-05	2.578e-07	3.245e-14

Table S3. Annual cumulative presence for 2015-2019 [0-5] – abiotic predictors. Spearman's rank correlation.

Predictor	Value	2015–2019
Average altitude (m)	Rho	−0.2444617
	S	6707606431
	p	<2.2e-16
Annual average humidity (%/100)	Rho	−0.2159552
	S	6.554e+09
	p	<2.2e-16
Surface of flowing water bodies (ha)	Rho	0.05768525
	S	5.079e+09
	p	0.001124
Surface of still water bodies (ha)	Rho	0.1838329
	S	4399112790
	p	<2.2e-16
Average temperature in spring (March–May) (degree C)	Rho	0.221787
	S	4194541752
	p	<2.2e-16
Average temperature in summer (June–August) (degree C)	Rho	0.2385037
	S	4104439048
	p	<2.2e-16
Average of maximal temperature for summer (degree C)	Rho	0.2250431
	S	4.177e+09
	p	<2.2e-16
Average of annual precipitations (mm/ sq m)	Rho	−0.2162213
	S	6555391139
	p	<2.2e-16

Table S4. Annual cumulative presence for 2015-2019 [0-5]—Host predictors. Spearman's rank correlation rho.

**Predictor**	**Value**	**2015–2019**
Human population	Rho	0.1966031
	S	4330281935
	p	<2.2e-16
Horse population	Rho	0.04486768
	S	5148130673
	p	0.01131
Fowl population	Rho	0.05209714
	S	5109164161
	p	0.003267
Sparrow probability	Rho	0.1444536
	S	4611366145
	p	2.539e-16
Dove probability	Rho	0.1997721
	S	4313201219
	p	<2.2e-16
Jackdaw probability	Rho	0.2509094
	S	4037572833
	p	<2.2e-16
Crow probability	Rho	0.1330848
	S	4672643376
	p	4.609e-14

Table S5. Spearman rank correlation coefficient (Rho values) for the human WNND cumulative presence (2015-2019) and the predictors, for the total number of human WNND cases for 2015-2019 and the predictors and the differences between them.

Predictor	Rho value—cumulative presences	Rho value—total number of infections	Difference
Average altitude (m)	−0.244461746	−0.244946792	0.000485046
Annual average humidity (%/100)	−0.215955233	−0.215575632	−0.000379602
Surface of flowing water bodies (ha)	0.057685252	0.057540266	0.000144986
Surface of still water bodies (ha)	0.183832916	0.184133839	−0.000300924
Average temperature in spring (March-May) (degree C)	0.221786966	0.223099567	−0.001312601
Average temperature in summer (June -August) (degree C)	0.238503714	0.239487194	−0.00098348
Average of maximal temperature for summer (degree C)	0.22504314	0.226349439	−0.001306299
Average of annual precipitations (mm/ sq m)	−0.216221254	−0.21585189	−0.000369364
Human population	0.196603099	0.197698904	−0.001095805
Horse population	0.044867681	0.044353655	0.000514026
Fowl population	0.052097135	0.052334385	−0.00023725
Sparrow population	0.144453566	0.144797857	−0.000344292
Dove population	0.199772083	0.200550074	−0.000777991
Jackdaw population	0.250909398	0.251570894	−0.000661496
Crow population	0.133084805	0.133882418	−0.000797613
